# Young workers’ perceptions about occupational carcinogens

**DOI:** 10.1177/10519815251382372

**Published:** 2025-10-31

**Authors:** Robert T Duffy, Anita Brobbey, Ela Rydz, Emma K Quinn, Sajjad S Fazel, Cheryl E Peters

**Affiliations:** 1 CAREX Canada, University of British Columbia, Vancouver, BC, Canada; 2 Department of Oncology, Cumming School of Medicine, University of Calgary, Calgary, AB, Canada; 3 Statistics Canada, Calgary, AB, Canada; 4 British Columbia Centre for Disease Control, Vancouver, BC, Canada; 5 BC Cancer Agency, Vancouver, BC, Canada

**Keywords:** awareness, cancer, hazards, work, youth

## Abstract

**Background:**

Young workers (≤25 years) face a well-documented increased risk of occupational injury, but little is known about their risk for occupational disease or how it compares to older workers, even though similar factors may contribute to both injuries and hazardous exposures.

**Objective:**

The objective of this mixed-methods study was to assess young workers’ ability to identify carcinogens and identify factors that may be indicative of a higher risk of occupational cancer.

**Methods:**

We conducted a survey of young workers in Canada and the United Kingdom via Prolific to assess knowledge, attitudes, and behaviours around carcinogenic exposures in the workplace. Participants were asked True/False (T/F) questions on factors affecting hazardous exposures, Likert-scale questions on workplace behaviours, and to identify carcinogens among various hazards. Scores were assigned based on ability to identify carcinogens, median scores were compared across demographics, occupational groupings, and responses. Participants were then recruited to participate in focus groups to discuss questions in further detail.

**Results:**

Median scores were lowest among participants in (1) retail and sales, and (2) agriculture, trades and manufacturing. Regardless of occupation, the ability to identify carcinogens was low. Median carcinogen scores were lower among incorrect T/F responses related to hazardous exposure. Many participants indicated a lack of knowledge regarding workplace hazards or how they may affect their health despite reporting receiving training.

**Conclusions:**

There are knowledge gaps by occupational groups that highlight a need for improvements to the delivery of training to young workers in the primary sector, manufacturing, and retail and sales.

## Background

The development of approximately 10,000 cancers annually in Canada has been attributed to workplace exposures to cancer-causing agents.^1^ The risk of young workers developing cancer can be caused by lasting effects of carcinogenic exposures and lifestyle habits acquired early in life.^2^ Early prevention strategies remain a key control in reducing disease onset within the population and must be tailored to at-risk subgroups, including young workers.^3,4^

Young workers (<25 years of age) may be at risk for the development of occupational disease, including cancer, for a variety of reasons. Adolescents and young adults have the longest length of time left to develop long latency diseases.^5^ Young workers are at higher risk of injury due to inexperience, lack of training, orientation, and supervision, lack of understanding of the workplace exposure to more dangerous jobs, and hesitancy to ask questions,^6^ factors that we hypothesize could similarly impact occupational disease risk. Young workers are exposed to various chemical, biological and physical exposures, and very few teenagers reported using suitable personal protective equipment (PPE).^7,8^ Furthermore, young workers in certain occupations are more likely to recognize their occupation as safe despite the presence of one or more hazardous tasks.^9^

A recent Canadian study from our team investigated the potential for carcinogenic exposures among young workers using the 2006 and 2016 Canadian Census data and found that the five occupations with the highest proportions of young workers included: (1) retail salesperson, clerks and cashiers, (2) chefs, cooks and servers, (3) sales and service occupations, (4) trades helpers, construction and transportation laborers, (5) occupations unique to primary sectors^.^^10^ Several of these industries (3, 4, and 5) have been highlighted by policy makers in Canada as high risk for health and safety.^11^ Additionally, young construction workers, farm workers, and outdoor workers were identified to be at a higher risk for occupational exposure to carcinogens based on (a) risk behaviour patterns (such as the lack or misuse of PPE or avoiding safety guidelines), (b) sectors with the large proportions of young workers, and (c) sectors with the highest number of occupational exposures.^10–12^ While sample evidence exists to demonstrate young workers are at a higher risk of injury and disease, there is still a general lack of understanding in Canada surrounding young workers’ understanding of their own hazardous exposures.

##  Objective

The objective of the present mixed-methods study was to test young workers’ ability to identify carcinogens, and to identify factors that may be indicative of a higher risk for the development of occupational cancer.

##  Methods

This study included a survey and a focus group component. The development of the survey for this study used a variety of previous study instruments, such as questions about PPE use and beliefs about carcinogens or other hazards, accompanied by a review of literature looking at barriers to occupational health and safety among young workers.^13–15^ Additionally, our team leads the CAREX Canada program of research, a national study in existence since 2007 that quantifies the prevalence and level of exposure to over 50 occupational carcinogens, and the exposures selected are the most prevalent exposures in workplaces in Canada, as verified additionally with our literature search.^16,17^ The study was approved by the Health Research Ethics Board of Alberta's Cancer Committee (HREBA.CC-19-0462).

In October of 2021, participants were invited to participate in a survey on young people's understanding about hazardous exposures in the workplace. Participants were required to be residents of Canada or the United Kingdom and be between the ages of 18–25 years. Additionally, participants had to indicate that they currently or have ever previously held a job (full-time or part-time) to be eligible to participate. Eligibility was not based on type or place of work. Participants who met the eligibility criteria were provided a questionnaire (see Appendix A) using Prolific.

Prolific is a platform that allows researchers to create and upload surveys and invite online participants.^18^ Respondents were provided financial compensation for their participation ($12.80 CDN/hr, for a survey of approximately 12 min to complete). The survey was run four times (Canada-female, Canada-male, UK-female, UK-male), restricting to males or females in each country to obtain approximately 50% male and female respondents. Prolific provides information on the number of participants meeting researchers’ demographics prior to survey launch so that determinations on sample size per survey could be made to meet the inclusion criteria.

### Statistical analysis

The survey consisted of three sections: understanding of work-related hazards and details regarding their present job, a module on sun safety knowledge and practices for those who worked outdoors, and a section on demographics (sex/gender, age, experience, location, and occupation). Data collected from the sun safety section was considered separately and is not included in the current evaluation. Jobs were classified using the National Occupational Classification (NOC) system into broad occupational categories (1-digit, *n* = 10).^19^ Descriptive statistics were restricted to those with complete data and were generated using The R Foundation for Statistical Computing (v4.1.2).^20^

The survey section on understanding of work-related hazards included true or false (T/F) questions regarding factors that affected hazardous exposure and workplace safety, and Likert scale questions regarding workplace hazards and personal protective equipment (PPE) use. The Likert scale was used to assess the strength with which the respondents agreed or disagreed with statements regarding their workplace experiences (see question 7 in the survey in Appendix A for the wording used). Respondents were also presented with 15 exposures (see question 9 in Appendix A), asked to identify which were known carcinogens, and were assigned a score based on their response (0–11). The 11 carcinogens included: arsenic, asbestos, benzene, diesel engine exhaust, formaldehyde, ionizing radiation, radon, second-hand smoke, silica dust, ultraviolet radiation, and wood dust. Those who correctly identified all known carcinogens in the list were given a score of 11. Carcinogen misidentifications were visualized in a heatmap that included all 10 occupational categories to observe which categories and carcinogens had the highest rates of misidentification. Median carcinogen scores were generated in R and compared using boxplots by occupational categories, sex, age, experience, and T/F questions, in order to identify risk factors for young workers. Responses to these questions were compared across collapsed occupational groupings to identify higher risk safety practices across occupations categories for further investigation.

Following the survey, focus group participants were restricted to Canadian respondents from the initial survey to aid in scheduling. When respondents participated in the survey, they had the option to indicate they were open to being contacted for follow up. A second survey, including focus group dates and times, was posted to the survey subgroup on Prolific, and closed when it reached 20 participants, as this is typically when qualitative research reaches saturation (beyond which new themes are unlikely to be identified).^21^ Participants were compensated £15 through Prolific after participating in the focus group.

Focus groups occurred through 60-min periods on Zoom over six separate days to accommodate participants’ availability and consisted of 2 or more participants. After being reminded of the focus group purpose and consenting to participate, participants were asked to answer a series of questions. Audio was recorded and transcribed by Zoom. Transcriptions were reviewed by MD, RD, and EQ to ensure accuracy.

Qualitative content analysis was used to study young workers’ perspectives about occupational exposures as collected in the focus groups. The analysis framework was conducted similarly to other media content analysis frameworks.^22,23^ Our analysis team (RD, EQ) independently identified themes developed *a priori*. Additional themes were added if necessary. Coding conflicts were resolved in a meeting, and any unresolved content was resolved by a third reviewer (CP). Interrater reliability for themes coded was 94%. Unique comments deemed valuable in the context of the focus groups were recorded separately and evaluated by coders. Coding categories of comments made by focus group participants were tabulated for percent of coverage in responses. Themes were assessed for frequency across responses and nodes per question.

##  Results

A total of 2050 participants consented to participate in the survey, and a total of 1839 provided complete, eligible survey responses (89.7% of total). Of the respondents, 48% were female and 49% were male (Table 1). Approximately 3% of the respondents identified as non-binary or two-spirit, self-identified, or preferred not to say. The highest proportion of respondents (45%) were between the ages of 18–21 and had less than one year of work experience. The highest proportion of respondents (40%) were working in sales and services occupations (NOC 6).

**Table 1.** Study sample characteristics.

Characteristic	Participants (*n* (%))
Country (*n* = 1839)	
Canada	1027 (55.9%)
United Kingdom	812 (44.2%)
Gender (*n* = 1839)	
Male	898 (48.8%)
Female	889 (48.3%)
Unspecified (Non-Binary, Two-Spirit, Self-Identify, Prefer not to say)	52 (2.83%)
Age (*n* = 1839)	
18–21	830 (45.1%)
22–23	506 (27.5%)
24–25	503 (27.4%)
Experience (*n* = 1839)	
Less than 1 year's work experience	829 (45.1%)
1–2 years’ work experience	550 (30.0%)
Advanced (2 + years)	460 (25.0%)
Broad Occupational Categories (*n* = 1839)	
Management occupations (0)	89 (4.84%)
Business, finance, and administration occupations (1)	270 (14.7%)
Natural and applied sciences and related occupations (2)	183 (9.95%)
Health occupations (3)	86 (4.67%)
Occupations in education, law and social, community and government (4)	250 (13.6%)
Occupations in art, culture, recreation, and sport (5)	87 (5.60%)
Sales and services occupations (6)	734 (39.9%)
Trades, transport and equipment operators and related occupations (7)	103 (5.60%)
Natural resources, agriculture, and related production occupations (8)	16 (0.87%)
Occupations in manufacturing and utilities (9)	21 (1.13%)

Table 2 indicates the proportion of responses that correctly identified each carcinogen, shown by country and in total. Exposures have been organized from highest level of misidentification to lowest. While there were not any substantial differences between respondents in the UK compared to Canada, respondents in Canada had higher proportions of correct responses for most (but not all) carcinogens. The overall mean carcinogen score was 4.93/11 (44.8%). Very few respondents (*n* = 50) achieved a perfect score of 11/11 (100%). Carcinogens that were most frequently misidentified (incorrect responses >50%) included arsenic, benzene, diesel engine exhaust, formaldehyde, radon, silica dust, and wood dust. The NOC groups with the lowest median carcinogen score (4) were retail/sales and trades/agriculture/manufacturing. The lowest median score (4) was found in respondents between the ages of 18–21 and with 2 + years of experience, though the finding of longer job tenure leading to lower knowledge scores was not statistically significant (*p* = 0.1497), indicating that job tenure did not influence participants’ knowledge about carcinogens. Respondents in the United Kingdom had a lower median score (4) than Canada (5), which is also reflected by the results shared in Table 2. There were no obvious differences in score by gender.

**Table 2.** Proportion of correctly and incorrectly identified carcinogenic exposures in Canada, the United Kingdom, and in total.

	Carcinogenic Exposures Identification		
Exposure	Canada, Correct Responses (% of 1027)	UK, Correct Responses (% of 812)	Overall, Correct Responses (% of 1839)
Wood dust	81 (7.89%)	65 (8.00%)	146 (7.94%)
Benzene	306 (29.80%)	170 (20.94%)	476 (25.9%)
Silica dust	291 (28.33%)	205 (25.25%)	496 (26.9%)
Formaldehyde	366 (35.64%)	175 (21.55%)	541 (29.4%)
Diesel engine exhaust	323 (31.45%)	277 (34.11%)	600 (32.6%)
Radon	457 (44.50%)	336 (41.38%)	793 (43.1%)
Arsenic	520 (50.63%)	358 (44.09%)	878 (47.7%)
Ionizing radiation	615 (59.88%)	534 (65.76%)	1149 (62.4%)
Asbestos	722 (70.30%)	577 (71.06%)	1299 (70.6%)
Ultraviolet radiation	769 (74.88%)	550 (67.73%)	1319 (73.3%)
Second-hand smoke	813 (79.16%)	559 (68.84%)	1372 (74.6%)

**Figure 1.**
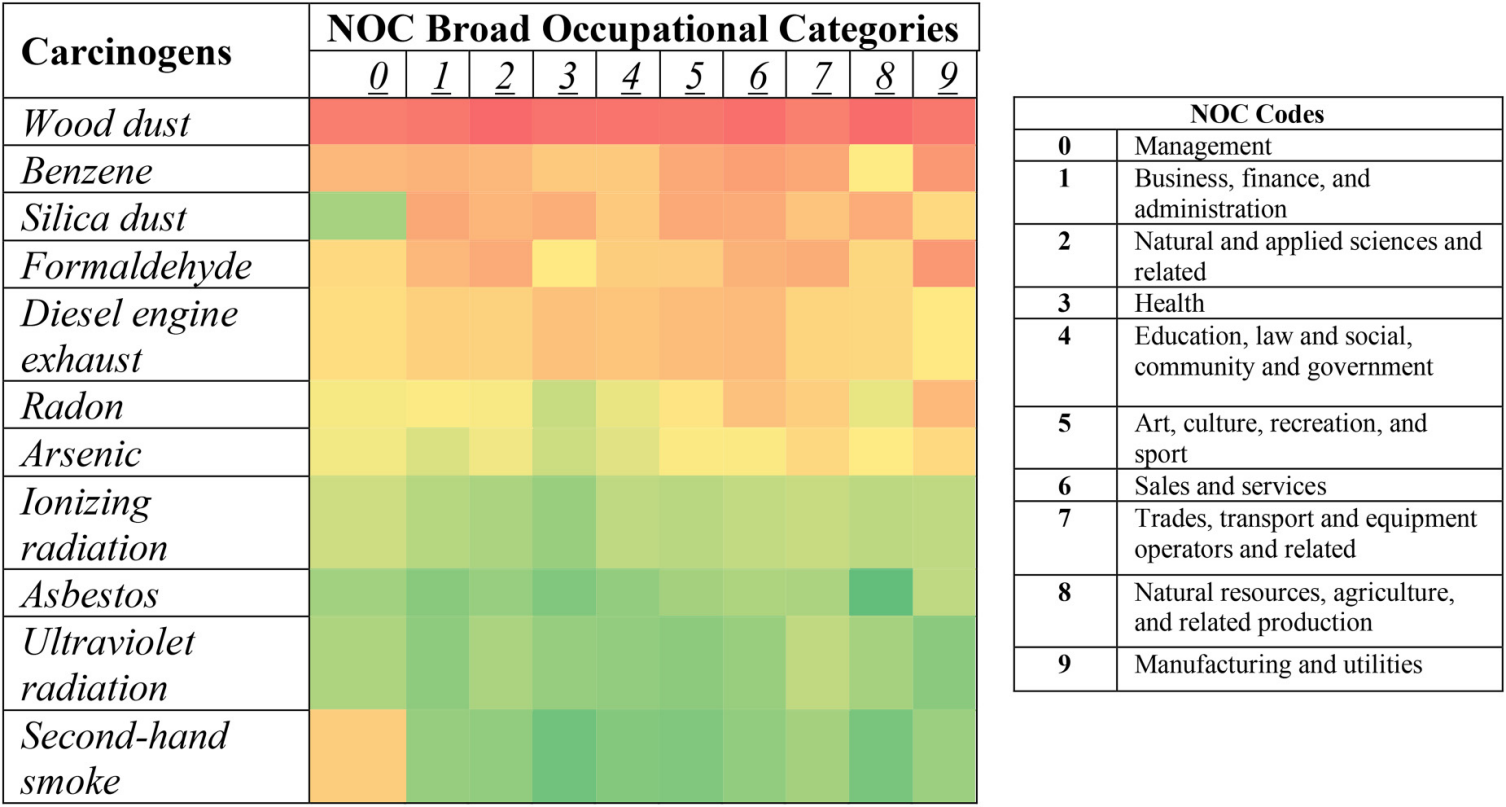
Heatmap of carcinogen identification by broad occupational category. *Numbers in the *Y*-axis correspond with the NOC broad occupational category codes. Cell colours indicate proportion of respondents with high (green) to low (red) carcinogen identification.

Figure 1 demonstrates that wood dust had the highest proportion of misidentification across all occupational categories. Benzene and formaldehyde had high proportions of misidentification in manufacturing and utilities operations. Second-hand smoke had the highest proportion of misidentification in management occupations.

Table 3 summarizes responses to all questions, including T/F, workplace practices, and PPE use. Approximately 63.5% of respondents agreed that they were informed about hazards in their workplace. Approximately 59.6% of respondents agreed that they were informed about how hazards affect their health, while 42.3% disagreed/were neutral that hazard information is accessible to them, and 42.1% disagreed/were neutral that hazard training is provided. Approximately 62.2% felt comfortable enough to refuse unsafe work. Stratifying responses by occupational grouping revealed some differences for workers in the retail and sales sectors and workers in office jobs. The retail and sales sector had the highest proportion of young workers reporting inaccessible hazard information (53.5%). Young workers in retail and sales, as well as in office jobs, had the highest rates of reported unavailable hazard training (43.3% and 44.8%, respectively). Fewer young workers in office jobs agreed that they were informed about the hazards in their workplace (59.1% vs. 63.5% overall) and how the hazards in their workplace affect their health (56.7% vs. 59.6% overall). For detailed results of analyses by occupational grouping for understanding of workplace hazards and reasons for lack of PPE, see Supplementary Tables 1 and 2.

**Table 3.** Proportion of responses to hazardous exposure true/false, understanding of risks and workplace practices, and PPE use questions.

Hazardous Exposure True/False		
Question	Correct Response	Incorrect Response
Chemicals and other hazardous substances can enter the body through breathing.	1814 (98.6%)	25 (1.35%)
Chemicals enter the body through accidental ingestion.	1775 (96.5%)	64 (3.50%)
Chemicals cannot enter the body through skin contact with contaminated surfaces.	1629 (88.6%)	210 (11.4%)
Chemicals enter the body through contact with spills and splashes	1710 (92.9%)	129 (7.01%)
Chemical gas and vapor in air can enter the body through the skin.	1378 (74.9%)	461 (25.1%)
Exposure to low levels of carcinogens (cancer-causing agents) is safe.	1379 (75.0%)	460 (25.0%)
Inhalation exposure to hazardous substances outdoors is negligible due to adequate ventilation.	1236 (67.2%)	603 (32.8%)
By law, workers have the right to refuse work if they believe it is unsafe.	1819 (98.9%)	20 (1.10%)

Knowledge and Attitudes		
Question	Agree	Disagree/Neutral
Informed about hazards at work	1168 (63.5%)	671 (36.5%)
Informed about how hazards affect health	1098 (59.6%)	741 (37.8%)
Hazard information is accessible	1064 (57.7%)	775 (42.3%)
Access to necessary PPE	1206 (65.2%)	633 (34.8%)
Access to other methods to reduce exposure	877 (47.5%)	962 (52.5%)
Hazard training is available	1067 (57.9%)	772 (42.1%)
Properly trained to use PPE	926 (50.3%)	913 (49.7%)
Confidence in proper PPE use	1183 (64.4%)	656 (35.6%)
Confidence in Protecting one-self from hazards	1319 (71.8%)	520 (28.2%)
Workplace is safe	1571 (85.3%)	268 (14.7%)
Supervisor supportive to questions regarding safety	1537 (83.3%)	302 (16.7%)
Coworkers use PPE adequately	1075 (58.4%)	764 (41.6%)
Feeling unprotected because coworkers cannot deploy methods to reduce exposure	208 (11.4%)	1631 (88.6%)
Making dangerous decisions at work	384 (20.8%)	1455 (79.2%)
Feeling comfortable to refuse unsafe work	1149 (62.2%)	690 (37.8%)

Personal Protective Equipment (PPE) Use		
Question	Agree	Disagree
I don’t think I’m at risk	999 (54.3%)	840 (45.6%)
I am concerned about what my coworkers would think	203 (11.0%)	1636 (88.9%)
Even if I am exposed, I don’t think I’m at risk of negative health outcomes	242 (13.2%)	1597 (87%)
Wearing PPE is uncomfortable or inconvenient	245 (13.3%)	1594 (86.6%)
I don’t have access to other equipment or methods that could help	229 (12.4%)	1610 (87.5%)
PPE is not provided	147 (7.99%)	1692 (92.0%)
Other*	706 (38.3%)	1133 (61.6%)
I don’t experience any hazardous exposures at work	89 (4.84%)	1750 (95.2%)

In general, approximately 50.3% of respondents agreed that they’ve been properly trained to use PPE, while approximately 52.5% of respondents felt as though they didn’t have access to other controls besides PPE to reduce their exposure. Fewer respondents in the retail and sales sector agreed that they had been properly trained to use PPE (46.6%). A higher proportion of workers in the agriculture, trades and manufacturing grouping were concerned about what their coworkers would think if they wore PPE (18.6% vs. 11.0% overall) and felt as though wearing PPE is uncomfortable or inconvenient (27.1% vs. 13.3% overall). The most common reason for PPE non-use in all groups was “I don’t think I’m at risk” (54.3%).

**Figure 2.**
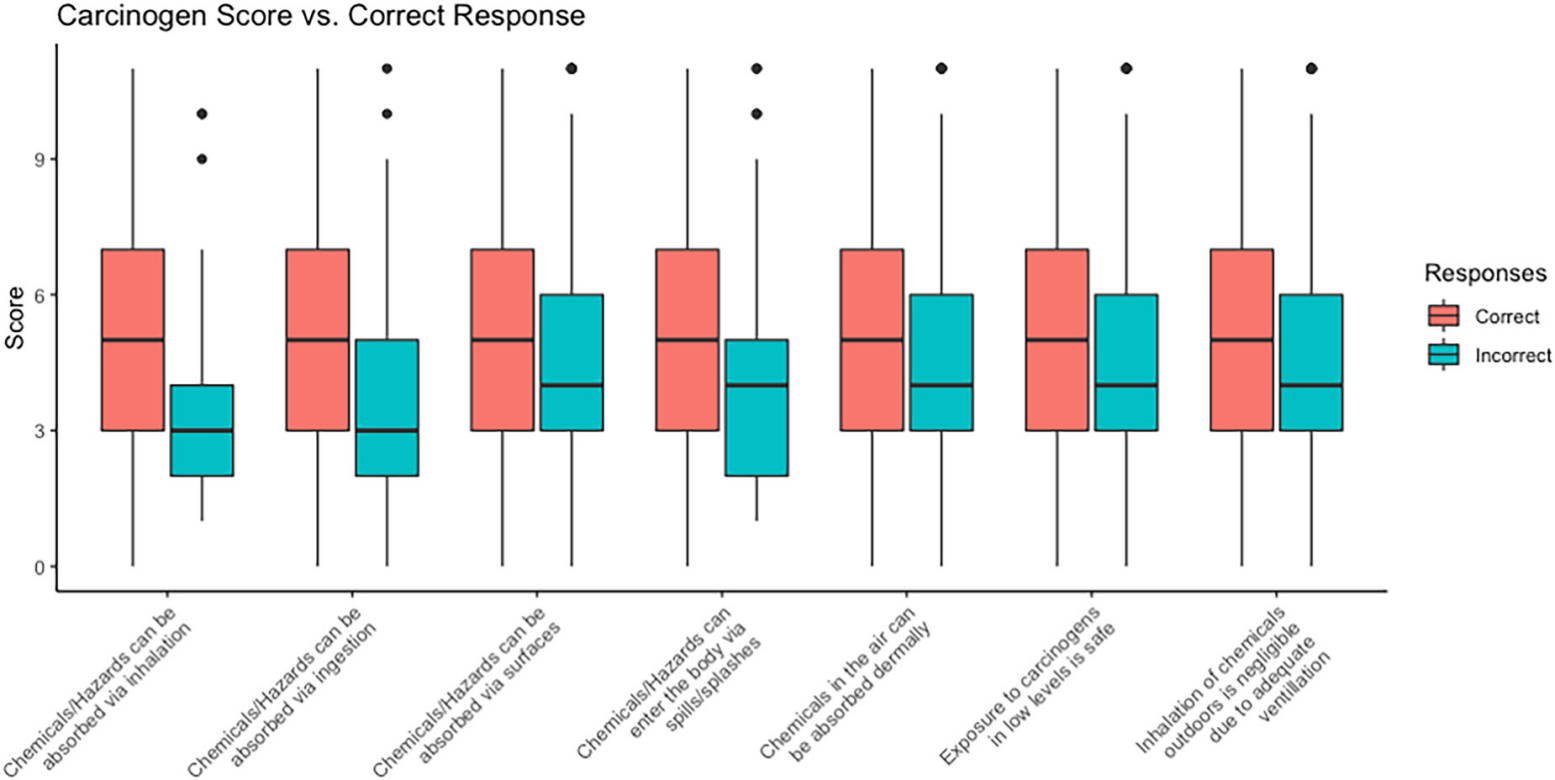
Grouped boxplots of carcinogen score vs. correct responses to true/false hazard exposure questions compared to carcinogen scores. Note: The line in each box indicates the median, and the top and bottom of the box represents an inter-quartile range (IQR).

More than 25% of respondents incorrectly responded to three true/false statements: dermal absorption of gas and vapours through the skin (24.2%), safety of low-level carcinogen exposure (24.2%), and exposure to hazards outdoors via inhalation (33.0%). Figure 2 depicts median carcinogen scores by responses to all T/F questions. Median carcinogen scores were lower among respondents who incorrectly answered T/F questions, with the lowest median carcinogen score (3) observed among those that were unaware of inhalation as a route of exposure for chemicals.

###  Focus group

Responses were coded for mention of themes, where n values of themes can be higher than the number of participants if mentioned more than once in response or question. When focus group participants were asked about their concerns in the workplace, the top responses were dust (*n* = 6, 22.2%) and bacteria or disease (*n* = 5, 18.5%), and carcinogens (*n* = 5, 18.5%). Nearly all responses indicated an increased presence of masks since the beginning of the COVID-19 pandemic (*n* = 16, 88.9%). Overall, many responses indicated feeling safe at work (*n* = 13, 24.1%) and having received good (*n* = 13, 24.1%) or mediocre (*n* = 7, 13.0%) training for the job. Although many reported receiving training, better training and new research were reported as recommendations (*n* = 4, 50.0%; *n* = 4, 50.0%).

Although some participants had experienced no adverse symptoms of exposure (*n* = 2, 13.3%) or were unsure (*n* = 5, 33.3%), dermal (*n* = 2, 13.3%), dizziness (*n* = 3, 20.0%), and respiratory symptoms (*n* = 3, 20.0%) were sometimes indicated. Responses to the question about whether their protection at work is adequate found most respondents were comfortable with current measures (*n* = 15, 44.1%). Friends (*n* = 3, 50.0%), doctors (*n* = 2, 33.3%), and parents (*n* = 1, 16.7%) were all asked questions about workplace exposures and protection. Barriers to engaging in PPE use included workplace culture (*n* = 7, 29.2%), discomfort (*n* = 7, 29.2%), time/convenience (*n* = 4, 16.7%), and unavailable (*n* = 3, 12.5%). Suggestions to reduce workplace exposures that were most frequent included better or more PPE (*n* = 14, 24.1%), addressing tasks and hazards (*n* = 11, 19.0%) or other physical harm/injury (*n* = 9, 15.5%). Participants reported finding employers (*n* = 8, 16.0%), governments (*n* = 8, 16.0%), and the internet (*n* = 8, 16.0%) as being the most trusting sources for information.

Seventeen responses were coded for unique statements. A selection of these are included here for context:

“If I or any other workers were to see us using a chemical or anything that we're supposed to have PPE for, and we're not. We would instantly stop each other and be like, “what are you doing? You should not be doing that so instantly”.

“I guess the workplace culture that's been developed as one that's takes workplace safety extremely seriously and if we're ever in a situation where someone's not doing the safe thing, **it's common practice for people to call that out** for people to I guess cease working when it's not safe or it's not safe for everyone around us”.

“There's a limit to how much you can kind of tolerate in terms of safety, right. If I want to wear a respirator all day every single, day, especially on days, where it's you know 40 degrees out, and I'm in the sun for 8 h you know, if I don't die of cancer, I'm gonna die of heat exhaustion, or something, right? This sort of limits to where you can kind of protect yourself”.

“I was like, this is not worth minimum wage”.

“After that I didn't take any real action afterwards mainly because it was a very short temporary job, and I was really just doing it for the money. So, I didn't care to have conditions improved and most other people don't burn as easily as I do”.

##  Discussion & conclusions

Our findings suggest a concerning lack of awareness surrounding many occupational carcinogens among young workers in both Canada and the UK. On average, respondents correctly identified less than half of the carcinogens. Median carcinogen scores suggest that young workers between the ages of 18–21 and with greater than 2 years of work experience have the lowest carcinogen awareness. Furthermore, young workers had the lowest median carcinogen scores in broad occupational groupings that included retail/sales and agriculture/trades/manufacturing, which are particularly alarming due to the high number of young workers and high risk of carcinogen exposures related to these occupations.^10^ Most frequently misidentified carcinogens included diesel engine exhaust, silica dust, arsenic, benzene, and radon, which are some of the top contributing carcinogens to the burden of occupational cancer in Canada,^1^ with many hundreds of thousands of Canadians exposed (i.e., not rare exposures). These findings are alarming for industries where these carcinogens are likely present, including occupations in the primary sector such as agriculture, as well as trades and manufacturing. Young workers were best able to identify asbestos, ultraviolet radiation, and second-hand smoke as carcinogens, which could speak to the targeted public education campaigns on cancer prevention that these exposures have received.

Despite the availability of hazard training, respondents still disagreed or felt neutral about whether they were being informed about hazards in their workplace or how the hazards they are exposed to affect their health. These findings are unsurprising, as workplace orientation and training has been found to be low for young and new workers.^24^ Almost half of respondents disagreed or felt neutral that they were being adequately trained to use their PPE. The work environment, task duration, restriction, interference, and comfort from PPE to complete a task were all discussed as factors that influenced the lack of PPE use by young workers during a workday.^25,26^

Occupations in retail and sales contribute to almost 30% of the young workforce.^10^ A major theme found among respondents for PPE non-use was the perception that their workplace was safe. It has been previously reported that young workers in the retail and services sector were exposed to various chemical, physical, and biological exposures, and very few teenagers reported using suitable PPE.^7^ Furthermore, workers in the retail and services sector may be more likely to recognize their occupation as safe despite being aware of the presence of hazardous tasks.^9^ There are legal requirements for safe work that also apply to young workers, and these include PPE provision and supervision, but it is less clear that these requirements are enforced regularly in Canada and the UK. More investigation is needed to understand the barriers to consistent PPE use when required.^15,27,28^ Of note, recruitment for this survey occurred during the COVID-19 pandemic, when mask mandates were implemented in both Canada and the UK. The use of PPE within this context could have influenced workers, especially those in sales and services, who would have not typically thought about using PPE to protect themselves.

Occupations in agriculture, trades, and manufacturing also contain a large proportion of young workers.^10^ Young workers in these sectors have already been found to be at high risk for injury.^13,29,30^ Hazard training and information availability were highest among respondents in the agriculture/trades/manufacturing group, while still maintaining one of the lowest median carcinogen scores, indicating potential gaps in hazard training delivery. It is possible that there are more trainings available in these sectors due to well-established hazards regularly encountered in these settings, but more research is required to elucidate the reasons behind why young people working in these settings are learning less about cancer-causing substances than is warranted.

A factor influencing health and safety practices among young workers may be related to fear. Although almost all workers (99%) were aware of their right to refuse unsafe work, many (37.5%) still did not feel completely comfortable in refusing it. One study found that most workers do not report injuries or hazards because they did not feel confident in reporting, did not want to cause a problem, and some feared losing their job as a result.^31^ Young workers aged 16–18 found that complaints were systematically discounted, and it often occurred in a gendered way.^23^ Many teenagers noted that accepting injury was “part of the job” and minor injuries/complaints were often silenced or dismissed by their supervisors.^32^ Women emphasized that their complaints were often disregarded by their superiors, while men avoided talking about their injuries to appear mature among their older co-workers.^32^

Management occupations were included as part of office-based jobs in the occupational groupings but may not be an appropriate categorization for specific occupational categories. For example, construction managers are classified in the NOC 0 grouping but are unlikely to cluster well with other office-type jobs in terms of tasks. Additional targeted recruitment would also allow for the expansion of broad occupational categories into the major and minor groupings for further analyses and would provide a better understanding of management occupation types. We did not detect substantial differences between survey respondents in Canada as compared to the UK, suggesting that the challenges facing young workers in these two countries are common.

Focus group responses suggest mixed understanding and experiences of workplace exposures and protective measures. Many participants reported feeling safe at work, and expressing that they received adequate training, however, when prompted by other questions specifically about exposures, symptoms, and recommendations, more details came out about potential hazards. Further mixed, some participants reported that they or their colleagues are likely to call each other out on unsafe practices, while one participant mentioned not caring about hazards because they needed the money from the job. Responses suggest that young workers seek information in their workplace, but when are unable to get useful responses, they feel comfortable searching for advice from family, government resources, or medical professionals. Further, participant responses suggest that improvement for workplace safety training, along with a shift in workplace culture could contribute to making their workplaces safer.

###  Limitations

This study has a number of strengths and limitations. Though the survey was developed based on other validated and reliable surveys, the final survey used for this study did not go through a rigorous validation process, which may raise some concerns about the reliability of the data collected. Using Prolific allowed for a quick recruitment of a large number of participants in a short period of time with a wider reach across Canada and the UK. Prolific has been found to be more reliable and produce higher quality data than other online research platforms.^33^ Furthermore, the anonymity of the survey allowed participants to be more willing to share sensitive information about their workplace without the fear of it impacting their job. Although the sample may be more demographically representative to other research platforms, online recruitment may not be representative to young workers of all industries nation-wide. Furthermore, information on socio-economic status, education, and marginalized populations were not collected from respondents. Purposely restricting the study population to obtain approximately 50% male- and female- identifying respondents did provide a better distribution of responses by gender-identifying groups; however, this still left a low number of participants in many groups, such as two-spirit and non-binary. Classifying occupations by NOC broad occupational code was beneficial to highlighting specific industries, but multiple occupational categories had small sample sizes, especially in NOC 8 (natural resources, agriculture, and related production occupations) and NOC 9 (occupations in manufacturing and utilities), which required the collapsing of several jobs into categories. Lower sample sizes in each category may affect the how representative the results are for the overall sector. Targeted recruitment in areas with lower numbers of young workers are recommended to reflect the distribution of young workers concentrated in NOC 6 (sales and services occupations). Finally, while we aimed to recruit approximately 2000 survey respondents to the quantitative phase of our study, this was based more on convenience and we did not calculate an *a priori* sample size; this does limit our ability to generalize to the broader population of young workers in Canada and the UK. However, we obtained many meaningful descriptive results that helped inform the qualitative phase of our study and have still provided a useful snapshot of young workers’ experiences in their workplaces. Finally, we did not assess the actual presence of carcinogenic exposures in the workplaces of our respondents, as we focused only on their knowledge of said risks. It is possible that some of the workers with demonstrated less knowledge about carcinogenic exposures were less exposed, but we are unable to interrogate this further, and future research to tie knowledge and actual exposure together would be worthwhile. Previous work from our team, using CAREX Canada data, has been published on the topic of typical exposures occurring in the jobs and industries typically held by young workers.^34^ This work demonstrated that there are no workplaces that entirely lack exposure to known or suspected carcinogens; even in retail and cashier jobs, some workers are exposed to solar radiation, polycyclic aromatic hydrocarbons, and night shift work. Jobs that are unique to the primary sector also experience similar risks, as well as exposure to diesel and gasoline engine exhausts and other chemical exposures.

These findings demonstrate that young workers lack awareness of some of the top contributing carcinogens to the burden of occupational cancer and are likely not receiving adequate hazard awareness training. This study highlights low awareness and insufficient training, suggesting there may be gaps in training materials and practices. Review of existing workplace training and safety guidelines, as well as identifying ways to encourage or incentivise adherence to safety recommendations may lead to a stronger understanding of workplace safety, and result in workers engaging in safer work practices. The broad occupational categories that scored the lowest in carcinogen identification were (1) sales/services, (2) trades, transports, and equipment operators, (3) natural resources, agriculture and related production, and (4) manufacturing and utilities. Our focus group findings suggest a concerning lack of awareness surrounding many occupational carcinogens among young workers in Canada. The carcinogen scoring system can continue to be tested against hazard-related questions to identify gaps and factors that are putting workers at an increased risk for injury.

Understanding the factors that affect young workers’ understanding of the hazards they may experience at work is essential to develop new guidelines to improve hazard awareness training for young workers, risk literacy, and subsequently health-protective practices. Further analysis into additional demographics of respondents is recommended to include information on other potentially equity-seeking groups (e.g., Indigenous and immigrant workers). Future research exploring the relationship between the presence of an occupational hazard and young workers awareness and identification of the hazards would play a key role in understanding the training gaps leading to poor occupational health and safety awareness.

## Supplemental Material

sj-docx-1-wor-10.1177_10519815251382372 - Supplemental material for Young workers’ perceptions about occupational carcinogens Supplemental material, sj-docx-1-wor-10.1177_10519815251382372 for Young workers’ perceptions about occupational carcinogens by Robert T Duffy, Anita Brobbey, Ela Rydz, Emma K Quinn, Sajjad S Fazel and Cheryl E Peters in WORK
