# Supplementary material for: Young workers’ perceptions about occupational carcinogens
Source: Work. 2025 Oct 31;83(4):1212–21. doi: 10.1177/10519815251382372 (PMC13053868; doi:10.1177/10519815251382372)
Supplement: sj-docx-1-wor-10.1177_10519815251382372 - Supplemental material for Young workers’ perceptions about occupational carcinogens [file sj-docx-1-wor-10.1177_10519815251382372.docx]

# Appendices:

*Appendix A.* Questionnaire

## Section 1: Workplace exposures

1. True or False:
   1. Chemicals and other hazardous substances can enter the body through breathing
   2. Chemicals enter the body through accidental ingestion
   3. Chemicals cannot enter the body through skin contact with contaminated surfaces
   4. Chemicals enter the body through contact with spills and splashes
   5. Chemical gas and vapor in air can enter the body through the skin
   6. Exposure to low levels of carcinogens (cancer-causing agents) is safe
   7. Inhalation exposure to hazardous substances outdoors is negligible due to adequate ventilation
   8. By law, workers have the right to refuse work due to unsafe conditions

For the following series of questions, we will ask you about hazards you encounter at work. Please think of the job you presently work the most hours at. If you are not presently employed, please think of the last job that you held. If you have never had a job, click **here** to skip to the next relevant question.

1. What is your job title? _______
2. What type of business or organization do you work in (e.g. construction, restaurant, government, etc.)? ________
3. How long have you been working/did you work in this job? ________years ____months
4. Would you describe your typical job/trade schedule as:
   1. Part-time
   2. Full-time
   3. More than full-time
   4. Not applicable (not presently employed)
5. Which of the following hazards do you come across at your workplace? Select all that apply:
   1. Biological (e.g. molds, fungi, bacteria, pathogens)
   2. Chemicals and fumes
   3. Fibres and dusts
   4. Metals
   5. Pesticides
   6. Radiation (not including sun exposure; we will ask about that later)
   7. Excess heat
   8. Falls and injuries
   9. Other (please specify): ______
   10. I do not come across these hazards at work
6. What are the top 5 specific hazards of concern at your workplace (e.g. asbestos, tripping and falling, exhaust fumes, etc.)? For each:
   1. How often are you exposed (rarely, sometimes, often)?
   2. How intense is the exposure (minimal, moderate, very intense)?
   3. What is your perception of the health risks associated to the exposures (low, moderate, high)?
7. What is your level of concern related to the following in your workplace?: (5 point scale, ranging from none, low, moderate, high to very high)
   1. Workplace exposure to **biological hazards** (e.g. molds, fungi, bacteria, viruses)
   2. Workplace exposure to **chemicals and fumes**
   3. Workplace exposure to **dusts and fibres**
   4. Workplace exposure to **metals**
   5. Workplace exposure to **pesticides**
   6. Workplace exposure to **radiation (not including from the sun)**
   7. Workplace exposure to **excess heat or cold**
   8. Work-related **falls and injuries**
   9. Workplace exposures to **cancer-causing substances**
8. For the following statements, select one of the following: strongly disagree, disagree, neutral, agree, or strongly agree
   1. I am informed about hazardous substances at work
   2. I am told about how hazardous substances can be harmful to my health
   3. Information about work hazards is easy to get at work
   4. I have access to the personal protective equipment I need to protect myself (e.g. gloves, respirator)
   5. I have access to other ways to reduce my exposure (such as local exhaust ventilation, wet cutting to reduce dust, adjusting my schedule to avoid hotter parts of the day, etc.)
   6. I am given the training I need to protect myself from hazards at work
   7. I am trained to use PPE properly
   8. I am confident that I can use PPE properly
   9. I am confident that I can protect myself at work from hazardous exposures
   10. It is important that you pay attention to this study. Please select “Strongly Disagree”
   11. My workplace feels safe
   12. My supervisor would be supportive if I had questions about my safety at work
   13. My coworkers use personal protective equipment and other ways to reduce their exposures, when available
   14. I feel unprotected because other workers are not able to or do not use methods to reduce their exposures and I am affected (e.g. they create a lot of dust in their work and I am exposed to it because I work nearby)
   15. I sometimes have to make decisions about my health and safety that could endanger me at work
   16. I would feel comfortable refusing work due to unsafe conditions
9. If you sometimes choose to not follow the safest procedures at work, why not? (select all that apply):
   1. I don’t think I’m at risk of exposure
   2. I am concerned about what my coworkers would think about me
   3. Even if I am exposed, I don’t think I’m at risk of negative health outcomes
   4. Wearing PPE is uncomfortable or inconvenient
   5. I don’t have access to other equipment or methods that could help reduce my exposure (such as fans for ventilation, changing the location of things I could trip over, etc.)
   6. PPE is not provided to me
   7. Other:_____
   8. I don’t experience any hazardous exposures at work
10. Now we would like to ask some questions about your sun exposure at work. At your present or most recent job, In the summer, on average, how many hours do you typically work outside per day?

- 0 hours
- 1 hour or less
- 2-3 hours
- 4-5 hours
- 6+ hour

1. Have you received sun safety training at this current job? Yes/No
   1. If yes, **briefly** describe what this training included: ____
2. Please indicate the extent that you agree with the following statements:
   1. At work, I am given the information I need to protect myself from solar ultraviolet radiation (UVR)
   2. Sun exposure on the job is a concern for me
   3. I think my sun exposure at work will increase my risk of skin cancer
   4. With the right equipment and supplies, I can reduce the amount of solar UVR I am exposed to while at work
   5. If I apply sunscreen, I will be adequately protected from the sun’s harmful UV rays
   6. I like looking tanned
3. For the following questions, think about what you do when you are outside AT WORK during the summer on a warm sunny day. Options: Never, rarely, sometimes, often, always
   1. How often do you wear SUNSCREEN?
   2. How often do you wear a SHIRT WITH SLEEVES that cover your shoulders?
   3. How often do you wear UV protection factor (UPF)-RATED CLOTHING?
   4. How often do you wear a WIDE-BRIMMED HAT?
   5. How often do you stay in the SHADE?
   6. How often do you wear SUNGLASSES?
   7. How often do you check the SOLAR UV INDEX?
   8. How often do you take REST BREAKS and SEEK AIR-CONDITIONED rest areas?
   9. How often do you SCHEDULE MOST STRENUOUS WORK TO COOLER TIMES OF THE DAY?
   10. How often do you REDUCE ACTIVITY to minimize overheating?
   11. How often do you CHECK HUMIDEX RATINGS?
   12. How often do you spend TIME IN THE SUN TO GET A TAN?
4. For each of the following statements, think about a typical, warm, sunny day when you are working outside during the summer. (Options: strongly disagree, disagree, neutral, agree, or strongly agree)
   It is convenient to:
   1. Wear SUNSCREEN
   2. Wear a SHIRT WITH SLEEVES that cover your shoulders
   3. Wear UV protection factor (UPF)-RATED CLOTHING
   4. Wear a WIDE-BRIMMED HAT
   5. Seek SHADE
   6. Wear SUNGLASSES
   7. Take REST BREAKS and SEEK AIR-CONDITIONED rest areas
   8. Schedule the MOST STRENUOUS WORK TO COOLER TIMES OF THE DAY
   9. REDUCE ACTIVITY to minimize overheating
5. When you work outdoors, does your employer provide you with: (Yes/No)
   1. Canopies or other types of shade structures?
   2. Sunscreen?
   3. Wide-brimmed hats?
   4. Neck flap or brim attachments for hats?
   5. Air-conditioned rest areas?
   6. The option to reschedule work to cooler times of the day?
   7. Drinking water?
6. What are the reasons that you don’t protect yourself from sun exposure while working outside? Choose all that apply:
   - I forget to (e.g. wear the protective gear, apply sunscreen, seek shade, etc.)
   - It’s too time-consuming (e.g. to apply sunscreen or to seek shade)
   - I don’t like the sticky consistency of sunscreen
   - It’s too hot or uncomfortable to wear long sleeved clothing
   - I don’t mind getting a tan
   - It’s too expensive
   - My coworkers don’t, so I don’t bother either
   - My employer does not provide any protective equipment
   - My employer does not support some of the methods of protection, such as taking a break or rescheduling work to cooler parts of the day
   - Other: _________
7. Based on your own general knowledge, which of the following substances are capable of causing cancer? Select all that apply

- Arsenic
- Asbestos
- Benzene
- Carbon monoxide
- Diesel engine exhaust
- Formaldehyde
- Ionizing radiation
- Lead
- Pesticides
- Radon
- Secondhand smoke
- Silica dust
- Ultraviolet radiation
- Wifi exposure
- Wood dust
- I do not think any of these cause cancer

True or false:

- 1. Exposure to solar ultraviolet radiation (UVR) has only short-term consequences.
  2. Exposure to solar UVR is only a concern on sunny days
  3. Exposure to solar UVR can cause cancer
  4. Skin cancer can be fatal
  5. If you apply sunscreen at the start of your outdoor activities, you do not need to reapply unless you go swimming or sweat excessively

1. What are the signs and health effects associated with solar UVR exposure? Select all that apply:

- Suntan
- Sun burn
- Freckles
- Skin cancer
- Cancer in or around the eyes
- Cataracts
- Wrinkling of the skin
- Sunspots
- Eye damage leading to impaired vision

1. Rank the following from the most protective to the least protective for skin cancer prevention:

- Seeking shade
- Applying sunscreen
- Wearing protective clothing, such as long-sleeved shirts and wide-brimmed hats
- Wearing sunglasses
- All provide the same degree of protectiveness

1. The hours of the day when you are most at risk from the sun's rays are:
   1. 8 a.m. to 12 p.m.
   2. 2 p.m. to 6 p.m.
   3. 11 a.m. to 3 p.m.
   4. None of the above
2. The UV Index is:
   1. A rating system for sunscreens
   2. A measure of the strength of the sun’s UV rays
   3. A rating system for protective clothing
   4. None of the above

### Personal characteristics

Now we need to understand some of your risk factors for skin cancer.

1. What is your natural hair colour?

- Red or light blonde
- Blonde
- Dark blonde or light brown
- Dark brown
- Black

1. What colour are your eyes?

- Light blue, light grey, or light green
- Blue, grey, or green
- Hazel or light brown
- Dark brown
- Brownish black

1. Which of the following skin colours best characterize you?:

- Skin colour is **light, pale white** (*Always burns, never tans*)
- Skin colour is **fair** (*Usually burns, tans with difficulty*)
- Skin colour is **white to olive** (*Sometimes develops a mild burn, gradually tan*)
- Skin colour is **olive to moderate brown** (*Rarely burns, tans with ease*)
- Skin colour is **brown to dark brown** (*Very rarely burns, tans very easily*)
- Skin colour is **very dark brown to black** (*Never burns, tans very easily*)

1. As a child, did you have more than one severe sunburn? (i.e., painful and/or blistering) Yes/No
2. Have you ever been told by your doctor that you have skin cancer? Yes/No
   1. If **YES**, what type? (e.g., melanoma, squamous cell, basal cell carcinoma. Leave blank if unknown.)
3. Has anyone in your IMMEDIATE family (mother, father, sister, brother, child) been told by a doctor that they have skin cancer? Yes/No/Don’t know/prefer not to say

## Section 2: Background and job information

1. Your gender:
   1. Cisgender/Transgender Man
   2. Cisgender/Transgender Woman
   3. Non-binary
   4. Two Spirit
   5. Prefer not to say
   6. Prefer to self-identify: _____
2. Your age (in years): _____years
3. In what province or territory do you work?
   1. Alberta
   2. British Columbia
   3. Manitoba
   4. Ontario
   5. Prince Edward Island
   6. Quebec
   7. New Brunswick
   8. Newfoundland and Labrador
   9. Northwest Territories
   10. Nova Scotia
   11. Nunavut
   12. Saskatchewan
   13. Yukon
4. What is the highest certificate, diploma, or degree that you have completed?
   1. Less than high school diploma or its equivalent (i.e., GED)
   2. High school diploma or its equivalent
   3. Some college, trade school, or university
   4. Completed college, trade school or university degree
   5. Prefer not to say
5. We are planning a second part to this study where we will be conducting interviews to ask more in-depth questions about your experiences as a young worker. **If you agree that we can contact you in the future to take part in this study**, please provide your name and email address here.

Name: ___________

Email: ____________

# Focus Group Interview – questions

** A list of potential carcinogens that may be present within the workplace will be shared with participants prior to and during the interview; list is appended below **

**General questions**

1. Can you describe, in detail, the tasks that you perform at your job?
2. Are you concerned about being exposed to carcinogens or other hazards at your workplace? Please describe in detail and refer to specific carcinogens, if possible.
   1. *Prompts: What are the sources of exposure? What tasks are you doing when you are most concerned? What do you think the impacts of the exposures will be?*
3. Can you describe, in as much detail as possible, a situation at your workplace when you feel like you are being exposed to a hazardous substance? Do you know what substance? What symptoms, if any, do or did you experience?
4. Is your employer currently providing you with training on work exposures and safety protocols?
   1. What does the training look like?
      1. *Prompts – types of activities included in the training, duration of training, level of enthusiasm by employers when administering training*
   2. Do you feel like the training and information provided is adequate? As in, do you feel as though you have the knowledge and tools necessary to protect yourself from exposures? And, do you feel safe at work? Please explain.
   3. Do you feel confident in refusing unsafe work? Can you share an experience where you felt pressured to work a task that was unsafe?
5. Describe the actions that you take at your workplace to reduce your exposure to carcinogenic substances. Please be specific. Refer to the list of carcinogens, if necessary.
   1. *Prompt – examples of actions or controls include personal protective equipment such as respirators, hardhats, wide-brimmed hats, gloves, long-sleeved clothing, chemical resistant clothing, etc; engineering controls like local exhaust ventilation, enclosed pressurized cabs, HEPA filters, using mechanized processes, etc.; administrative controls like scheduling, cleaning and maintaining equipment, using wet-cutting methods, etc.*
6. If you were going to learn about the health effects of solar UVR exposure, or some of the other carcinogens that you are exposed to at your workplace, **where would you go** for this sort of information? Please name all the places.
   1. *Examples, if requested: Internet, unions, government, medical office, research agencies, newspapers, television, libraries, social media, radio, safety data sheets (SDSs)*
7. Please list all the persons, agencies, or groups that you would **trust** for information on workplace exposures and their possible associated health effects.
   1. *Examples, if requested: Health Canada, doctor, workplace health and safety committees, NIOSH, CDC, Canadian Auto Workers, etc.*
8. Could you describe, in as much detail as possible, a situation when you have talked to someone **AT** your workplace about solar UVR or other carcinogenic exposures?
   1. *Prompts: how did they react, did you feel supported, what actions did you take afterwards?*
9. Could you describe, in as much detail as possible, a situation when you have talked to someone **OUTSIDE OF** your workplace about solar UVR or other carcinogenic exposures?
   1. *Prompt/example: Have you ever talked to your doctor about being exposed to carcinogens or other chemical substances at your workplace? How did they react, did you feel supported, what actions did you take afterwards?*
10. What are barriers to using personal protective equipment within the workplace? How about administrative and engineering controls? Please describe in detail.
    1. *Examples, if requested: workplace culture, uncomfortable, expensive, not provided by workplace, takes too much time, doesn’t seem like a big deal not to use it, etc.*
11. Has the COVID-19 pandemic changed your perspective on PPE use at work? If so, in what ways?
12. Do you have any recommendations on what health researchers, health and safety organizations, or policy makers can do to address carcinogen exposure at your workplace?

**Questions specific for outdoor workers (solar UVR):**

1. How do you feel about being exposed to UVR at your workplace?
   1. Are you concerned about the exposure, and potential health effects? Why or why not?
2. Does your employer’s training on workplace health and safety include information on sun safety?
   1. What type of information is presented? Do you feel like adequate information was provided? Please discuss.
3. Do you feel adequately protected from solar UVR in the workplace? Why or why not?
4. Do you have any recommendations on what health researchers, health and safety organizations, or policy makers can do to address UVR exposure at your workplace
